# Solid Electrochemiluminescence Sensor by Immobilization of Emitter Ruthenium(II)tris(bipyridine) in Bipolar Silica Nanochannel Film for Sensitive Detection of Oxalate in Serum and Urine

**DOI:** 10.3390/nano14050390

**Published:** 2024-02-20

**Authors:** Ruliang Yu, Yujiao Zhao, Jiyang Liu

**Affiliations:** School of Chemistry and Chemical Engineering, Zhejiang Sci-Tech University, Hangzhou 310018, China; 202230107422@mails.zstu.edu.cn (R.Y.); 2023211001071@mails.zstu.edu.cn (Y.Z.)

**Keywords:** solid electrochemiluminescence sensor, immobilized emitter, bipolar silica nanochannel film, co-reactant, oxalate

## Abstract

Convenient and highly sensitive detection of oxalate ions in body fluids is of crucial significance for disease prevention, diagnosis, and monitoring of treatment effectiveness. Establishing a simple solid-state electrochemiluminescence (ECL) sensing system for highly sensitive detection of oxalate ions is highly desirable. In this work, a solid ECL sensor was fabricated by immobilizing the commonly used emitter ruthenium(II)tris(bipyridine) (Ru(bpy)_3_^2+^) on a double-layered bipolar silica nanochannel array film (bp-SNA)-modified electrode, enabling sensitive detection of oxalate ions in serum or urine samples. Cost-effective and readily available indium tin oxide (ITO) was used as the supporting electrode. Convenient fabrication of multiple negatively charged SNA (n-SNA)-modified ITO electrodes was achieved through the one-step Stöber solution growth method. Subsequently, a positive outer layer film (p-SNA) was rapidly prepared using an electrochemical-assisted self-assembly method. The double-layered bipolar silica nanochannel array film achieved stable immobilization of Ru(bpy)_3_^2+^ on the electrode surface, facilitated by the electrostatic adsorption of Ru(bpy)_3_^2+^ by n-SNA and the electrostatic repulsion by p-SNA. Utilizing oxalate ions as a co-reactant for Ru(bpy)_3_^2+^, combined with the electrostatic enrichment of oxalate ions by p-SNA, the constructed sensor enabled highly sensitive detection of oxalate ions ranging from 1 nM to 25 μM and from 25 μM to 1 mM, with a detection limit (LOD) of 0.8 nM. The fabricated ECL sensor exhibited high selectivity and good stability, making it suitable for ECL detection of oxalate ions in serum and urine samples.

## 1. Introduction

The detection of oxalate ions in body fluids is of crucial significance for disease prevention, diagnosis, and monitoring of treatment effectiveness [1,2,3,4]. Taking gout as an example, oxalate ions are one of the main pathogenic factors in this disease [5,6]. In patients with gout, abnormal metabolism or insufficient excretion of oxalate ions leads to an elevated level of oxalate ions in the blood, triggering the deposition of oxalate crystals in the joints, causing arthritis. The concentration of oxalate ions in urine is also associated with the formation of urinary stones [7,8,9]. As oxalate ions are primarily excreted through the kidneys, the level of oxalate ions in the blood is closely related to renal function. High concentrations of urinary oxalate ions may also contribute to the formation of oxalate ion stones. Additionally, oxalate ions can serve as a biomarker for other diseases, aiding in early detection and intervention. For instance, an elevated level of oxalate ions in the blood is related to the occurrence and development of metabolic diseases, such as metabolic syndrome and diabetes [10]. Some studies suggest that high oxalate ion levels may be associated with the occurrence and development of cardiovascular diseases [11]. Therefore, the detection of oxalate ions in blood and urine is of great significance for monitoring renal function, assessing the risk of cardiovascular diseases, diagnosing gout, and detecting urinary stone formation. Currently available methods for oxalate ion detection include enzymatic methods [12], high-performance liquid chromatography (HPLC) [13], liquid chromatography-tandem mass spectrometry (LC-MS/MS) [14,15], electrochemical methods [16], fluorescence spectroscopy [17], ultraviolet-visible spectroscopy (UV-Vis) [18], and capillary electrophoresis (CE) [19]. Enzymatic analysis exhibits high selectivity but is limited by the susceptibility of enzyme activity to environmental factors. HPLC and LC-MS/MS offer high resolution and sensitivity but are expensive and complex, making them unsuitable for rapid scenarios. Electrochemical methods can be used for real-time monitoring but have high sample processing requirements. UV-Vis detection is simple and fast, suitable for transparent solutions, but may suffer from low sensitivity for colored samples. Fluorescence spectroscopy has high sensitivity but is suspectable for background fluorescence. CE is suitable for small-volume samples, providing good separation, but it involves complex operations. The development of a convenient and highly sensitive method holds significant importance for oxalate ion detection in body fluid samples.

In recent years, electrochemiluminescence (ECL) sensors have gained significant attention for detecting oxalate ions [20]. ECL technology combines principles of electrochemistry and luminescence, where the core principle involves the generation of chemiluminescent substances on the electrode surface through electrochemical reactions, enabling the detection of target analytes [21]. ECL exhibits advantages such as high sensitivity, wide linear range, and low detection limit [22,23,24]. Additionally, it has the potential for real-time monitoring [25]. It has been proven that oxalate ions can serve as the target analyte for ECL detection, as they can serve as the co-reactant of the commonly used ECL emitter, ruthenium(II)tris(bipyridine) (Ru(bpy)_3_^2+^) [26]. Compared to a solution-phase ECL sensor, immobilizing an ECL emitter on the electrode surface to construct a solid-state ECL system enhances the stability and signal intensity of the sensor, making it more suitable for various practical applications [27,28]. On the one hand, the immobilization of ECL-emitting molecules prevents their dissolution, drift, or loss during operation, ensuring the stability of the luminescent material and thereby enhancing the reliability of the sensor. Moreover, solid-state ECL systems contribute to increased signal intensity [29]. On the other hand, the reaction between the ECL emitter and the electrode is facilitated by tightly fixing the ECL emitter to the electrode surface, improving sensitivity. Thus, in comparison with solution-phase ECL sensors, solid-state ECL systems eliminate the need for additional probes, simplifying experimental procedures and enhancing the operability of experiments, particularly in on-site detection or portable instruments, thus increasing the practicality of the sensor [30]. Therefore, establishing a simple solid-state ECL sensing system for highly sensitive detection of oxalate ions in complex biological samples such as serum or urine is highly desirable.

Porous materials are renowned for their unique structure and properties, characterized by a large surface area, high porosity, tunable pore size and structure, excellent diffusivity, and conductivity [31,32,33,34]. These features make porous materials widely applicable in various fields such as energy storage, catalysis, adsorption, separation, sensing, and medicine [35,36,37]. Utilizing porous molecular sieve film to modify electrodes provides an effective method for constructing solid-state ECL systems. Among them, silica nanochannel array film (SNA) has attracted considerable attention [29,30]. Specifically, SNA is prepared through a sol–gel process using surfactant micelles (SM) as templates, and it exhibits not only high mechanical and thermal stability and biocompatibility but also advantages such as uniform distribution of nanochannels (typically, 2–3 nm), tunable pore sizes (enlarged by introducing pore-expanders), and a high specific surface area [38,39,40,41]. Due to its ultra-small nanochannel array, SNA demonstrates a molecular-level sieving effect [42,43]. On one hand, high-density ultra-small nanochannels can prevent large molecules such as proteins, DNA, and particles from entering, exhibiting a significant size sieving effect [44,45,46,47]. Thus, SNA-modified electrodes demonstrate excellent anti-interference and anti-fouling capabilities, making them suitable for the direct detection of small molecules in complex matrix samples. On the other hand, the silica structure of SNA is rich in silanol groups. The low p*K*_a_ (~2) allows it to ionize in commonly used buffer media, forming a negatively charged surface [48,49]. Consequently, SNA-modified electrodes exhibit charge-based selectivity, electrostatically repelling negatively charged small molecules while attracting positively charged ones. Thus, SNA can electrostatically adsorb commonly used ECL emitters with a positive charge [50]. Combined with its high specific surface area, this characteristic enhances the immobilization of ECL emitters in solid-state ECL sensing systems. Therefore, SNA-modified electrodes hold the potential to construct high-performance solid ECL sensors with outstanding anti-interference/anti-fouling capabilities as well as high detection sensitivity, suitable for the detection of oxalate ions in complex samples.

In this study, a solid ECL sensor was fabricated based on stable immobilization of ECL emitters by integrating double-layered bipolar SNA with opposite charge properties on inexpensive and readily available electrodes, enabling highly sensitive detection of oxalate ions in serum and urine. Inexpensive and readily available indium tin oxide (ITO) electrodes were employed as supporting electrodes. Large-area negatively charged (n-SNA) was first prepared in a one-step process using the Stöber solution growth method [51]. Multiple n-SNA-modified electrodes (n-SNA/ITO) could be obtained in a single procedure. Subsequently, employing electrochemical-assisted self-assembly (EASA), amino-modified SNA with a positive charge (p-SNA) was grown on the surface of n-SNA/ITO electrodes [52]. The resulting bipolar SNA-modified electrode (bp-SNA/ITO) exhibited an electrostatic nanocage array structure. By mechanically stirring the common ECL emitter, Ru(bpy)_3_^2+^, into the inner n-SNA channels, stable immobilization of Ru(bpy)_3_^2+^ was achieved. This was facilitated by the electrostatic attraction of the negatively charged inner channels of n-SNA and the electrostatic repulsion of the positively charged outer channels of p-SNA. Due to the positive charge of the outer film, a high concentration of negatively charged oxalate ions could be enriched in the channels of p-SNA, serving as the co-reactant and enhancing the ECL intensity of the system. Combining the interference-resistant and anti-fouling properties of SNA, bp-SNA/ITO demonstrated promising applications in the ECL detection of bioorganic small molecules.

## 2. Materials and Methods

### 2.1. Chemicals and Materials

Sodium oxalate (CH_3_COONa), sodium dihydrogen phosphate dihydrate (NaH_2_PO_4_·2H_2_O), disodium hydrogen phosphate dodecahydrate (Na_2_HPO_4_·12H_2_O), cetyltrimethylammonium bromide (CTAB), tetraethyl orthosilicate (TEOS), potassium hexacyanoferrate(III) (K_3_[Fe(CN)_6_]), potassium hydrogen phthalate (KHP), 3-aminopropyltriethoxysilane (APTES), and hexaammineruthenium(III) chloride (Ru(NH_3_)_6_Cl_3_) were obtained from Aladdin Biochemical Technology Co., Ltd. (Shanghai, China). Ruthenium(II) tris(bipyridine) chloride hexahydrate (Ru(bpy)_3_Cl_2_·6H_2_O) was purchased from Sigma-Aldrich (Shanghai, China). Sodium nitrate (NaNO_3_) and anhydrous ethanol (99.8%) were acquired from Gaojing Fine Chemical Co., Ltd. (Hangzhou, China). Acetone and concentrated hydrochloric acid (HCl, 38%) were obtained from Shuanglin Reagent Co., Ltd. (Hangzhou, China). All solutions used in the experiments were prepared using ultrapure water (18.2 MΩ·cm). Indium tin oxide (ITO) conductive glass (sheet resistance <17 Ω/sq, ITO thickness: 100 ± 20 nm) was purchased from Zhuhai Kawei Optoelectronics Technology Co., Ltd. (Zhuhai, China). Prior to use, the ITO electrodes were immersed overnight in a NaOH aqueous solution (1 M), followed by ultrasonic cleaning in acetone, ethanol, and water solutions, respectively.

### 2.2. Characterizations and Instrumentations

All ECL and electrochemical tests were conducted using the conventional three-electrode system. Typically, ITO- or SNA-modified ITO served as the working electrode, a platinum wire or platinum foil served as the counter electrode, and silver/AgCl (with saturated KCl as the internal reference solution) served as the reference electrode. ECL tests were performed on the MPI-E II instrument (Xi’an Rui Mai Analytical Instrument Co., Ltd., Xi’an, China). Cyclic voltammetry (CV) was carried out using a three-electrode system on an Autolab electrochemical workstation (PGSTAT302N, Metrohm, Zofingen, Switzerland) with a scan rate of 50 mV/s. Morphological characterization was conducted through transmission electron microscopy (TEM, HT7700, Hitachi, Japan) and scanning electron microscopy (SEM, ULTRA 55, Carl Zeiss, Jena, Germany). To prepare TEM samples, the bilayer film was scraped off the electrode with a knife, placed in anhydrous ethanol, and sonicated for 2 h to ensure uniform dispersion. The dispersed solution was dropped onto a copper grid, air-dried naturally, and observed by TEM with an accelerating voltage of 200 kV. To obtain SEM samples, a knife was used to create a scratch on the back of the bp-SNA/ITO electrode, gently breaking it to expose a complete cross-section. After the gold coating, the sample was observed by SEM with an accelerating voltage of 5 kV.

### 2.3. Preparation of an n-SNA- or bp-SNA-Modified ITO Electrode

Using the Stöber solution growth method, n-SNA was grown on ITO [51]. CTAB (160 mg) was added to a mixed solution of water (70 mL) and ethanol (30 mL). After complete dissolution of CTAB, ammonia solution (10%, 100 μL) and TEOS (80 μL) were added, and the mixture was stirred until no bubbles were present. The ITO electrode was then immersed in the solution and sealed for a 24 h reaction at 60 °C. After thorough washing of the obtained electrode, it was aged for 12 h at 100 °C. Subsequently, it was stirred in a 0.1 M HCl-ethanol solution for 5 min to remove the surfactant micelles, resulting in a negatively charged SNA-modified electrode, denoted as the n-SNA/ITO electrode.

The EASA method was employed to continue the growth of the p-SNA film on the n-SNA/ITO electrode [52,53,54]. Specifically, CTAB (1.585 g) was added to a mixed solution of NaNO_3_ (0.1 M, pH = 2.6, 20 mL) and ethanol (20 mL) and stirred until dissolved. APTES (318 μL) was then added, and the pH was adjusted to 2.97 using HCl. Then, TEOS (2732 μL) was added, and the mixture was stirred for 2.5 h. Afterwards, the n-SNA/ITO electrode was immersed and served as the working electrode, and a constant current (j = −0.7 mA/cm^2^) was applied for 15 s. After extensive washing with ultrapure water, the obtained electrode was aged for 12 h at 120 °C. It was then stirred in a 0.1 M HCl-ethanol solution for 5 min to remove the SM, resulting in a bipolar film-modified electrode, denoted as the bp-SNA/ITO electrode.

### 2.4. Immobilization of Ru(bpy)_3_^2+^ on bp-SNA/ITO

The bp-SNA/ITO electrode was immersed in a solution of Ru(bpy)_3_^2+^ (1 mM). The solution was stirred for 20 min to achieve the immobilization of Ru(bpy)_3_^2+^ on the bp-SNA/ITO electrode. The resulting electrode was referred to as the Ru@bp-SNA/ITO electrode.

### 2.5. ECL Detection of Oxalate Ions 

The Ru@bp-SNA/ITO electrode was immersed in PBS solution (0.01 M, pH = 7.4) containing varying concentrations of oxalate ions for 2 min. Subsequently, a continuous CV scan was employed to trigger the ECL process. The CV potential ranged from 0 to 1.4 V, with a scan rate of 100 mV/s. The photomultiplier tube voltage for ECL was set at 550 V. For real sample analysis, the determination of oxalate ions in fetal bovine serum (diluted 50 times) or urine (diluted 10 times) was performed using the standard addition method.

## 3. Results and Discussion

### 3.1. The Strategy for the Construction of Solid-State ECL Sensors

**Figure 1:**
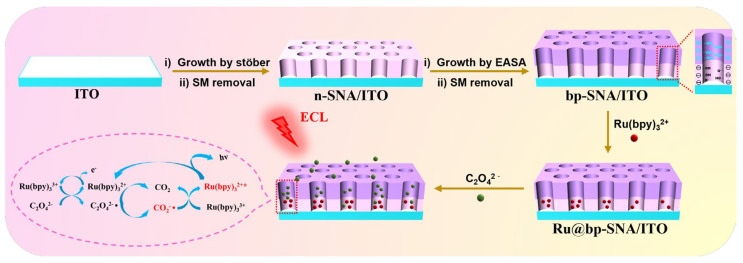
Schematic illustration for the preparation of Ru@bp-SNA/ITO and the sensing mechanism for C_2_O_4_^2−^.

Efficient and stable immobilization of ECL emitters on the electrode surface is crucial to ensuring the high performance of solid-phase ECL sensors. As shown in Figure 1, a dual-layered SNA with different charge properties is constructed on the electrode surface, which is employed to immobilize the commonly used ECL emitter Ru(bpy)_3_^2+^ for the detection of oxalate ions. In this study, a readily available indium tin oxide (ITO) electrode is chosen as the supporting electrode. ITO electrode, also known as ITO conductive glass, is prepared by sputtering ITO thin films on glass, exhibiting good conductivity and stable binding with SNA by forming Si-O-Sn or Si-O-In bonds. Currently, the preparation of the dual-layered bp-SNA is mainly achieved through sol–gel reactions of siloxane under the template control of surfactant micelles. The commonly used methods include Stöber solution growth and the electrochemical-assisted self-assembly method (EASA). The former involves regulating the sol–gel reaction of the siloxane precursor in ethanol/ammonia medium, resulting in a long synthesis time but offering the possibility of preparing large-area SNA-modified films in one step. The latter involves applying a negative voltage or current to the electrode, electrolyzing water on the electrode surface in situ, generating OH^−^ and pH gradients, and achieving rapid preparation of SNA, usually within seconds to tens of seconds. 

As illustrated in Figure 1, a negatively charged SNA (n-SNA) is grown on an ITO electrode using the Stöber solution growth method. Taking advantage of the easy cutting of ITO, this process allows for the one-time preparation of multiple n-SNA-modified electrodes (n-SNA/ITO). Subsequently, by introducing amino-functionalized siloxane and using the EASA method, an amino-modified positively charged SNA (p-SNA) is grown on an n-SNA/ITO electrode. After removing the micelles, an open nanochannel array is obtained, resulting in a bipolar SNA-modified electrode (bp-SNA/ITO). Thus, the bp-SNA/ITO electrode has an inner layer of n-SNA and an outer layer of p-SNA, forming an electrostatic nanocage structure. When the bp-SNA/ITO electrode is immersed in a solution of Ru(bpy)_3_^2+^, the positively charged Ru(bpy)_3_^2+^ probes are confined to the inner layer of n-SNA channels through mechanical stirring, achieving immobilization of the ECL emitter (Ru@bp-SNA/ITO). When the Ru@bp-SNA/ITO electrode is immersed in a solution containing oxalate ions, ECL detection of oxalate ions can be realized as they can serve as co-reactants for Ru(bpy)_3_^2+^.

### 3.2. Characterization of the bp-SNA/ITO Electrode

**Figure 2:**
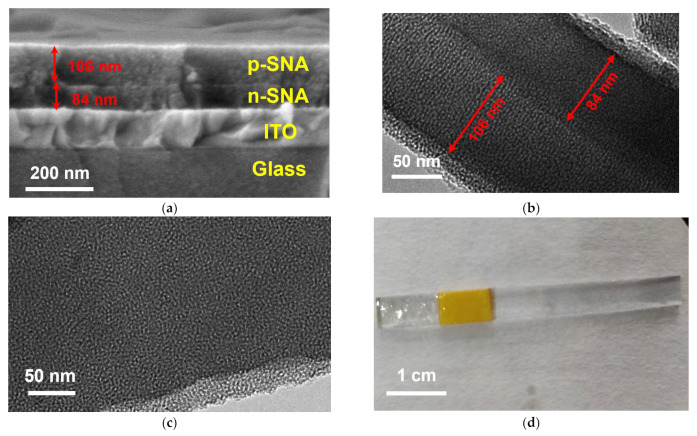
(**a**) Cross-sectional SEM image of the bp-SNA/ITO electrode. (**b**) Cross-sectional TEM image of bp-SNA. (**c**) Top-view TEM image of bp-SNA. (**d**) A digital picture of the as-prepared bp-SNA/ITO electrode. The yellow area represents insulating tape. The left section of the insulating tape is a bp-SNA-modified electrode.

The morphology of the constructed bp-SNA/ITO electrode was characterized through scanning electron microscopy (SEM), as shown in Figure 2a. The bp-SNA/ITO electrode exhibits a four-layer structure, consisting of the glass layer and indium tin oxide layer of the ITO electrode at the bottom, followed by the n-SNA layer and the p-SNA layer. The thickness of the n-SNA layer and the p-SNA layer is 84 nm and 106 nm, respectively. Figure 2b presents a transmission electron microscopy (TEM) image of the cross-section of bp-SNA, clearly showing the presence of a two-layer structure of nanochannels. The thicknesses of the two films are consistent with the SEM characterization results. In Figure 2c, a TEM top-view of the bp-SNA/ITO electrode reveals that the film is composed of numerous microporous structures. There is no collapse or rupture within the observed range, and SNA exhibits a relatively uniform pore size (approximately, 2–3 nm). Undoubtedly, this ultra-small nanochannel film structure possesses a high specific surface area, suitable for the following immobilization of Ru(bpy)_3_^2+^. Figure 2d shows a digital image of the as-prepared bp-SNA/ITO electrode. Following the modification with bp-SNA, the electrode remains transparent.

**Figure 3:**
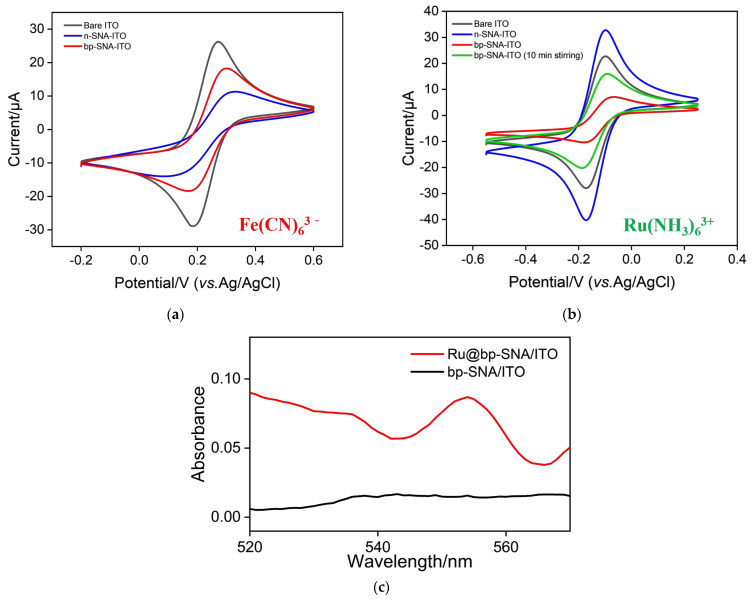
(**a**,**b**) CV curves of the ITO, n-SNA/ITO, and bp-SNA/ITO electrodes in 0.05 M KHP containing 0.5 mM K_3_Fe(CN)_6_ ((**a**), pH 4) or 0.5 mM Ru(NH_3_)_6_Cl_3_ ((**b**), pH 4). The scan rate was 50 mV/s. (**c**) The UV image of Ru@bp-SNA/ITO and bp-SNA/ITO electrodes.

To validate the integrity of the SNA film and charge-based selective permeability, the electrochemical signals of the negatively charged redox probe, Fe(CN)_6_^3−^, were measured on different electrodes. The CV curves are depicted in Figure 3a. Compared to the ITO electrode, the Fe(CN)_6_^3−^ signal recorded on the n-SNA/ITO electrode decreased. This is attributed to the presence of abundant silanol groups (p*K*_a_~2) on the nanochannel surface of n-SNA, which ionize in the measurement medium. The phenomenon generates a negative surface charge that results in electrostatic repulsion towards Fe(CN)_6_^3−^, leading to a significant reduction in the peak current signal. When the p-SNA is further modified on the electrode, amino groups in the nanochannels can electrostatically enrich Fe(CN)_6_^3−^, resulting in an increase in peak current. Combining the electrostatic repulsion from the inner n-SNA, the Fe(CN)_6_^3−^ signal measured on the bp-SNA/ITO electrode is slightly lower than that on the ITO electrode but higher than that on the n-SNA/ITO electrode. 

When the standard redox probe is replaced with the positively charged Ru(NH_3_)_6_^3+^, the results are shown in Figure 3b. Due to the negative nature of the channels, n-SNA/ITO exhibits electrostatic adsorption towards the positively charged Ru(NH_3_)_6_^3+^, leading to a higher peak current signal compared to the ITO electrode. In contrast, the bp-SNA/ITO electrode, owing to the electrostatic repulsion of the outer p-SNA to Ru(NH_3_)_6_^3+^, shows a decrease in signal. When the bp-SNA/ITO electrode is immersed in a stirred solution of Ru(NH_3_)_6_^3+^ for 10 min, the signal of Ru(NH_3_)_6_^3+^ on the electrode significantly increases. Thus, during stirring, Ru(NH_3_)_6_^3+^ can overcome the electrostatic repulsion of p-SNA and be enriched by the inner n-SNA.

Figure 3c displays the ultraviolet-visible (UV-Vis) spectra of the bp-SNA/ITO and Ru@bp-SNA/ITO electrodes. Compared to the bp-SNA/ITO electrode, the electrode containing Ru(bpy)_3_^2+^ exhibits a characteristic peak of Ru(bpy)_3_^2+^ between 550 and 560 nm, indicating the successful confinement of the Ru(bpy)_3_^2+^ probe within the electrode.

### 3.3. ECL Process of Ru(bpy)_3_^2+^ Using Oxalate Ions as Co-Reactants

**Figure 4:**
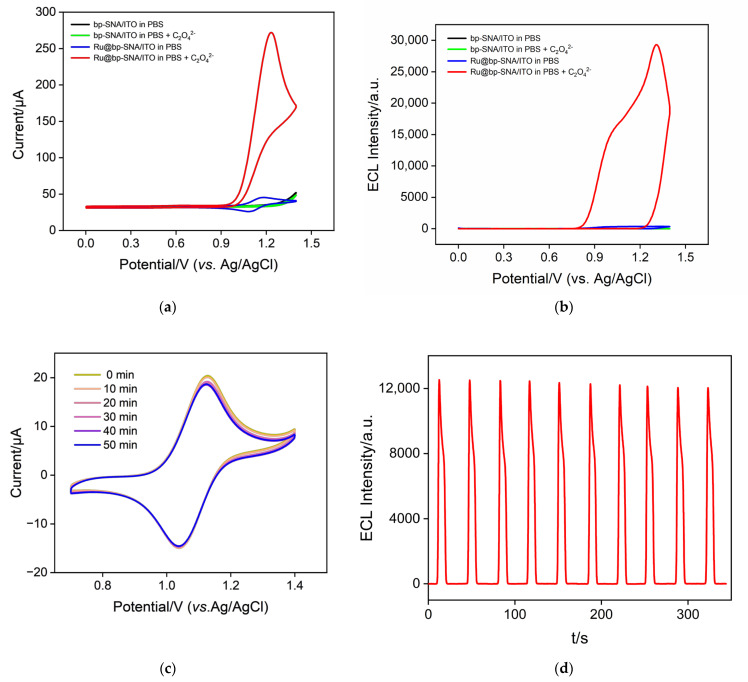
CV curves (**a**) and the corresponding ECL responses (**b**) obtained on different electrodes in 0.01 M PBS (pH 4) in the absence or presence of 1 mM C_2_O_4_^2−^. (**c**) CV curves obtained on the Ru@bp-SNA/ITO electrode after it was immersed in 0.01 M PBS (pH 7.4) for different times. The scan rate was 50 mV/s. (**d**) ECL curves obtained on Ru@bp-SNA/ITO electrodes in 0.01 M PBS (pH 7.4) containing C_2_O_4_^2−^ for 10 cycles.

Figure 4a shows the electrochemical signals of the bp-SNA//ITO electrode in different solutions. In the PBS medium, there is no apparent Faradaic signal observed on the electrode. The addition of C_2_O_4_^2−^ to the solution also does not result in a significant Faradaic signal on the electrode, indicating that oxalate ions themselves do not exhibit electrochemical activity within the measured potential range. The curve of the Ru@bp-SNA/ITO electrode in PBS solution shows redox peaks attributed to Ru(bpy)_3_^2+^ ions. When the Ru@bp-SNA/ITO electrode is immersed in a solution containing C_2_O_4_^2−^, a noticeable enhancement in peak current signal is observed, demonstrating the catalytic effect of C_2_O_4_^2−^ on the electrochemical process of Ru(bpy)_3_^2+^. Figure 4b represents the corresponding ECL signals during the CV scans. As seen, the Ru@bp-SNA/ITO electrode exhibits a high ECL signal in the presence of C_2_O_4_^2−^, attributed to the role of oxalate ions as co-reactants to facilitate the ECL of Ru(bpy)_3_^2+^. As depicted in Figure 1, Ru(bpy)_3_^2+^ loses an electron on the electrode to become Ru(bpy)_3_^3+^, which is subsequently reduced by C_2_O_4_^2−^ in the solution to regenerate Ru(bpy)_3_^2+^ and oxalate radical (C_2_O_4_^−^). The C_2_O_4_^−^ then decomposes to form carbonate radical anion (CO_2_^·−^) and carbon dioxide. CO_2_^−^ reacts with Ru(bpy)_3_^3+^ to generate carbon dioxide and the excited state (Ru(bpy)_3_^2+*^), which, in turn, returns to the ground state, releasing energy and producing the ECL signal.

### 3.4. Stability of the Immobilized Ru(bpy)_3_^2+^

The stability of the probe is a critical factor in maintaining the high performance and reliability of solid-state ECL sensors under different conditions. The stability of the Ru(bpy)_3_^2+^ emitter immobilized on the Ru@bp-SNA/ITO electrode surface was investigated by measuring the electrochemical and ECL signals of the immobilized Ru(bpy)_3_^2+^. As shown in Figure 4c, even after continuous immersion in the solution for 50 min, the oxidation-reduction signal of Ru(bpy)_3_^2+^ still maintained over 95% of the initial signal. When the electrode was subjected to 10 consecutive CV scans to measure its ECL signal, the ECL signal remained highly stable, with a relative standard deviation (RSD) of 1.5% in ECL intensity (Figure 4d). This demonstrated the high stability of Ru(bpy)_3_^2+^ immobilized on the electrode surface. This stability is attributed to the electrostatic cage structure of the bipolar SNA film, which exerts a confining effect on Ru(bpy)_3_^2+^. On one hand, Ru(bpy)_3_^2+^ experiences electrostatic adsorption from the inner n-SNA and, simultaneously, electrostatic repulsion from the outer p-SNA. The combined dual electrostatic effects contribute to the high stability of the immobilized Ru(bpy)_3_^2+^.

**Figure 5:**
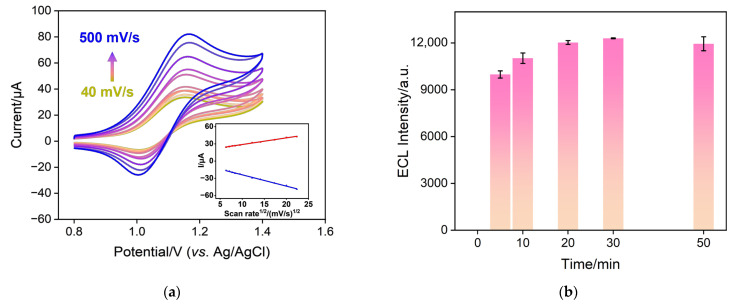

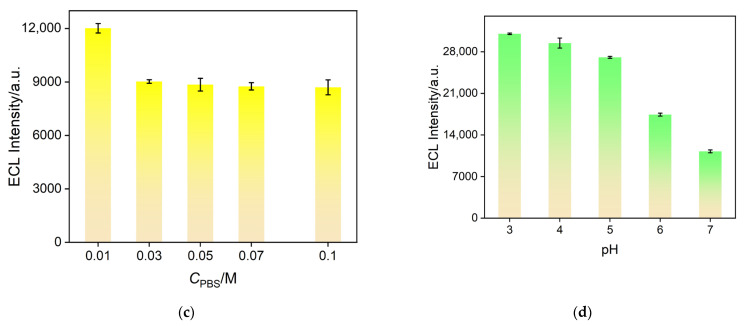
(**a**) CV curves obtained on the Ru@bp-SNA/ITO electrode in 0.01 M PBS (pH 7.4) containing 1 mM C_2_O_4_^2−^ at different scan rate. The scan rate used for CV curves from top to bottom was 500, 400, 300, 250, 200, 150, 100, 70, 40 mV/s, respectively. The inset is the linear relationship between peak current and the square root of the scan rate. (**b**) ECL responses obtained from Ru@bp-SNA/ITO electrodes in 0.01 M PBS (pH 7.4) prepared when Ru(bpy)_3_^2+^ was enriched for different times. (**c**) ECL responses obtained from Ru@bp-SNA/ITO electrodes in different concentrations of PBS containing 1 mM C_2_O_4_^2−^. (**d**) ECL responses obtained from Ru@bp-SNA/ITO electrodes in different pH values of PBS containing 1 mM C_2_O_4_^2−^.

To investigate the type of electrochemical reaction occurring on the electrode surface with Ru(bpy)_3_^2+^, CV scans were conducted on the Ru@bp-SNA/ITO electrode at various scan rates, as depicted in Figure 5a. It can be observed that as the scan rate increases, both the anodic and cathodic peak currents of Ru(bpy)_3_^2+^ increase. The anodic peak current (*I*_a_) and cathodic peak current (*I*_c_) exhibit a well-defined linear relationship with the square root of the scan rate (*ν*^1/2^) (*I*_a_ = 1.16 *ν*^1/2^ + 17.2, *R*^2^ = 0.996; *I*_c_ = −1.97 *ν*^1/2^–4.21, *R*^2^ = 0.997, inset in Figure 5a). This phenomenon confirms a diffusion-controlled electrochemical process.

### 3.5. Optimization Conditions for Ru@bp-SNA/ITO Construction and Oxalate Detection

To achieve maximum detection sensitivity, optimization was performed for the construction conditions of the Ru@bp-SNA/ITO electrode and detection, including the enrichment time of Ru(bpy)_3_^2+^, the concentration, and the pH of the detection electrolyte. Figure 5b illustrates the ECL signals obtained for electrodes with different Ru(bpy)_3_^2+^ enrichment times. Thanks to the bipolarity of SNA and its dual electrostatic interactions with Ru(bpy)_3_^2+^, the Ru(bpy)_3_^2+^ probe can be effectively confined to the inner n-SNA film solely through mechanical stirring. With an increase in enrichment time, the ECL signal of the electrode increases. After 20 min of enrichment, the ECL signal tends to stabilize, indicating that the confined Ru(bpy)_3_^2+^ has reached saturation. Thus, 20 min was chosen as the optimal enrichment time for Ru(bpy)_3_^2+^.

Figure 5c represents the optimization of the PBS concentration in the electrolyte for oxalate detection. As shown, the ECL signal of the electrode decreases with an increase in PBS concentration. This is attributed to the reduction in double layer thickness with an increase in solution concentration. The amino and silanol groups in the channels of the bipolar film provide charge-selective permeability to SNA. An increase in PBS concentration reduces the thickness of the double electric layer between the channels, weakening the enrichment effect of C_2_O_4_^2−^ and the confinement effect on Ru(bpy)_3_^2+^. However, excessively low ion strength weakens the buffering effect and lowers the conductivity of the solution, thereby reducing signal intensity and stability. A PBS concentration of 0.01 M was ultimately selected as the detection electrolyte. Figure 5d depicts the optimization of solution pH for detection. It can be observed that the ECL signal of the electrode significantly increases as the pH decreases from 7 to 5. This may be attributed to the increase in the positive charge of the outer p-SNA film as the pH decreases, enhancing its enrichment effect on C_2_O_4_^2−^ and thereby amplifying the ECL signal. Subsequently, as the pH continues to increase, the ECL signal continues to rise, but the magnitude of the increase is smaller. Thus, a slightly acidic pH of 5 was ultimately selected as the optimal detection pH.

### 3.6. ECL Detection of Oxalate

**Figure 6:**
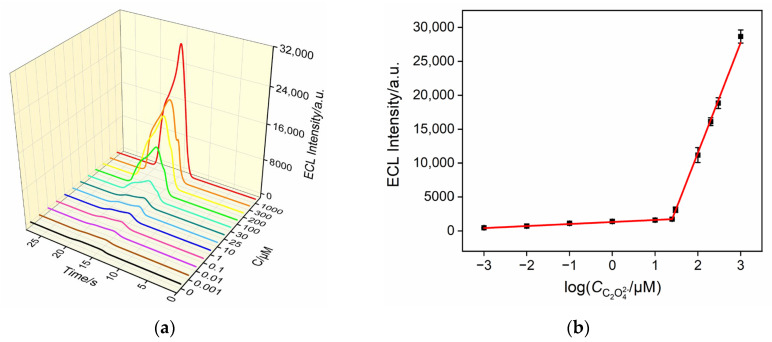
(**a**) ECL responses of the Ru@bp-SNA/ITO electrode in 0.01 M PBS (pH 4) containing various concentrations of C_2_O_4_^2−^. (**b**) The corresponding calibration curves.

Under the optimized conditions, Ru@bp-SNA/ITO electrodes were immersed in sodium oxalate solutions of varying concentrations, and the ECL signal was measured. As shown in Figure 6a, the ECL intensity gradually increases with the increasing concentration of oxalate ions. A good linear correlation is observed between the ECL intensity (*I*_ECL_) of the electrode and the logarithm of the concentration of the oxalate ion (log*C*). For oxalate ion concentrations ranging from 1 nM to 25 μM, the corresponding linear regression equation is *I*_ECL_ = 299log*C* + 1316 (*R*^2^ = 0.998). When the concentration of the oxalate ion is from 25 μM to 1 mM, the linear regression equation is *I*_ECL_ = 16,235log*C* – 20,964 (*R*^2^ = 0.999). The calculated limit of detection (LOD) based on three times the signal-to-noise ratio (S/N = 3) is 0.8 nM.

### 3.7. Selectivity, Reproducibility, and Stability of Detection

**Figure 7:**
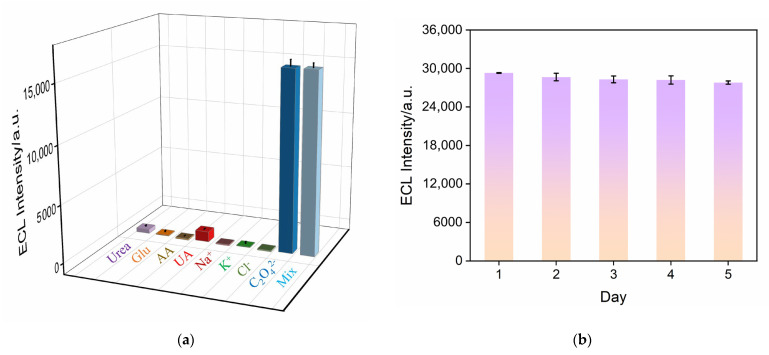
(**a**) ECL responses obtained on the Ru@bp-SNA/ITO electrode in 0.01 M PBS (pH 4) containing 200 μM C_2_O_4_^2−^ and other substances (400 μM) or their mixture. (**b**) ECL responses obtained on Ru@bp-SNA/ITO electrodes after storage for different days in 0.01 M PBS (pH 4) containing 1 mM C_2_O_4_^2−^. PMT = 550 V. The scan rate was 100 mV/s.

To meet the requirements of practical sample detection, the selectivity of the Ru@bp-SNA/ITO electrode for detecting oxalate ions was investigated. Common interfering substances found in actual samples were selected for examination. For example, glucose (Glu) is present in serum samples and urea in urine, and commonly encountered oxidative substances such as ascorbic acid (AA) and uric acid (UA) often coexist with oxalate ions in urine. K^+^, Na^+^, Cl^−^, and others are among the most common inorganic ions in real samples. Figure 7a presents the ECL signals of the Ru@bp-SNA/ITO electrode in solutions or mixtures of the above substances, as well as the oxalate ion solution. The concentrations of these potential interfering substances were twice those of the oxalate ions. It can be observed that, except for uric acid, no significant ECL signals were detected in solutions containing the other substances. Even with uric acid at a concentration twice that of oxalate ions (0.4 mM, higher than the normal concentration of urea in adult blood and urine), the ECL signal detected was still less than 10% of that in the oxalate ion solution, demonstrating high detection selectivity.

To investigate the reproducibility of the fabricated electrode, five electrodes were prepared in the same batch. The RSD for detecting oxalate ions was 2.1%. Figure 7b shows the stability of the electrode after different days of storage. After 5 days of storage, the ECL signal remained over 95% of the initial electrode signal, demonstrating the good stability of the electrode.

### 3.8. Real Sample Analysis

To evaluate the capability of the constructed sensor for real sample analysis, the standard addition method was employed to determine the oxalate content in bovine serum samples (diluted 50 times) or urine samples (diluted 10 times). As shown in Table 1, the detection exhibited good recovery rates (91.05% to 109.0%) and low RSD values (1.2% to 4.4%), demonstrating good accuracy for real sample analysis.

## 4. Conclusions

In summary, a solid ECL sensor was fabricated through stable immobilization of Ru(bpy)_3_^2+^ by constructing a two-layered and bipolar silica nanochannel film (bp-SNA)-modified electrode, which can realize sensitive detection of oxalate ions. The bp-SNA comprises an inner layer with a negative charge (n-SNA) and an outer layer with a positive charge (p-SNA). Due to the electrostatic attraction of the inner layer of n-SNA to Ru(bpy)_3_^2+^ and the electrostatic repulsion of the outer layer of p-SNA, the Ru(bpy)_3_^2+^ probe can be stably immobilized on the electrode surface through simple mechanical stirring. In comparison to conventional ECL analysis systems with solution-based emitters, the solid-state ECL sensor developed in this study is characterized by its simple operation. Thanks to the positive charge of the outer layer of p-SNA, which also facilitates the enrichment of oxalate ions, the sensor exhibits high sensitivity. Owing to its high selectivity, good reproductivity, and stability, the fabricated sensor holds great potential for highly sensitive ECL detection of oxalate ions in body fluids.

## Figures and Tables

**Table 1:** Detection of C_2_O_4_^2−^ in serum or urine samples.

Samples	Added (μM)	Found (μM)	Recovery (%)	RSD (%)
Serum ^a^	0.1000	0.1090	109.0	1.2
	100.0	91.05	91.05	3.2
	1000	1072	107.2	4.4
Urine ^b^	0.0000	24.48	-	4.3
	100.0	115.7	91.19	1.3
	200.0	235.2	105.4	3.2
	1000	1090	106.6	3.5

^a^ The fetal bovine serum was diluted by a factor of 50 using 0.01 M PBS (pH 4). ^b^ The urine was diluted by a factor of 10 using 0.01 M PBS (pH 4).

## Data Availability

The data presented in this study are available on request from the corresponding author.
